# Travel distance to rifampicin-resistant tuberculosis treatment and its impact on loss to follow-up: the importance of continued RR-TB treatment decentralization in South Africa

**DOI:** 10.1186/s12889-024-17924-0

**Published:** 2024-02-23

**Authors:** Katherine C. McNabb, Alanna J. Bergman, Amita Patil, Kelly Lowensen, Nomusa Mthimkhulu, Chakra Budhathoki, Nancy Perrin, Jason E. Farley

**Affiliations:** 1 Johns Hopkins University School of Nursing, 525 N Wolfe St, Baltimore, MD 21205 USA; 2 Johns Hopkins Center for Infectious Disease and Nursing Innovation, Baltimore, MD USA; 3 Johns Hopkins Center for Infectious Disease and Nursing Innovation, Port Shepstone, Republic of South Africa; 4 Johns Hopkins TB Research Advancement Center, Baltimore, MD USA

**Keywords:** Loss to follow-up, Care retention, Care engagement, Tuberculosis, Rifampicin-resistant tuberculosis, Multidrug-resistant tuberculosis, HIV, South Africa

## Abstract

**Background:**

Understanding why patients experience loss to follow-up (LTFU) is essential for TB control. This analysis examines the impact of travel distance to RR-TB treatment on LTFU, which has yet to be analyzed within South Africa.

**Methods:**

We retrospectively analyzed 1436 patients treated for RR-TB at ten South African public hospitals. We linked patients to their residential ward using data reported to NHLS and maps available from the Municipal Demarcation Board. Travel distance was calculated from each patient’s ward centroid to their RR-TB treatment site using the *georoute* command in Stata. The relationship between LTFU and travel distance was modeled using multivariable logistic regression.

**Results:**

Among 1436 participants, 75.6% successfully completed treatment and 24.4% were LTFU. The median travel distance was 40.96 km (IQR: 17.12, 63.49). A travel distance > 60 km increased odds of LTFU by 91% (*p* = 0.001) when adjusting for HIV status, age, sex, education level, employment status, residential locale, treatment regimen, and treatment site.

**Conclusion:**

People living in KwaZulu-Natal and Eastern Cape travel long distances to receive RR-TB care, placing them at increased risk for LTFU. Policies that bring RR-TB treatment closer to patients, such as further decentralization to PHCs, are necessary to improve RR-TB outcomes.

Loss to follow-up (LTFU) from rifampicin-resistant tuberculosis care (RR-TB) is challenging current TB control efforts [1]. This incomplete treatment can lead to further resistance to available anti-TB drugs and the transmission of drug-resistant TB (DR-TB) to contacts [2]. Of those who are LTFU, the majority die within just a few years of leaving treatment, if not reengaged in care [3]. In South Africa (SA), recent studies indicate that between 6 and 16% of people undergoing RR-TB treatment are LTFU [4, 5], meaning that they have missed greater than two consecutive months of treatment [6]. Given the estimated 21,000 incident cases of drug-resistant TB that occur annually in South Africa [7], addressing LTFU is a public health imperative.

Despite the dire implications of LTFU, effective interventions to reduce it are lacking. Known risk factors for LTFU in SA include male sex, younger age, HIV status, alcohol use, substance use, and site of TB disease, while protective factors include stable housing and steady employment [8]. Unfortunately, most of these risk factors are either non-modifiable or the distal results of complex societal problems that are challenging for the TB control program to address immediately. Identifying new, modifiable, and actionable risk factors is imperative to decreasing LTFU and improving RR-TB treatment outcomes.

One potential risk factor for LTFU that has yet to be thoroughly investigated is distance traveled to receive RR-TB treatment. Within KwaZulu-Natal (KZN) and Eastern Cape (EC) provinces, RR-TB treatment is initiated and monitored at centralized and decentralized facilities that provide access to the specialized medications and expertise needed to treat RR-TB [9, 10], requiring some patients to travel long distances to initiate and/or receive RR-TB care [11]. Although RR-TB treatment decentralization has been ongoing since 2011 [9], participants in more recent qualitative studies still express that transportation time and cost are barriers to care retention [12]. Travel distance is an obvious common factor underlying these findings; however, no prior studies have analyzed its impact on RR-TB care retention in SA. Accordingly, this analysis aims to evaluate the relationship between travel distance to RR-TB treatment on LTFU across 10 RR-TB treatment sites in KZN and EC provinces in SA.

## Methods

### Parent study

This retrospective analysis used secondary data from a cluster-randomized trial investigating the impact of a nurse case management intervention on RR-TB outcomes in the KZN and EC provinces of SA. People aged 13 years and older receiving RR-TB care at one of the 10 participating South African TB hospitals between 2014 through 2020 who provided informed consent were consecutively enrolled in the parent study. People with extensively drug-resistant TB (XDR-TB) or pre-XDR-TB (as defined by the World Health Organization (WHO) during the study timeframe) were excluded [13]. The 10 participating South African TB hospitals were selected in collaboration with the provincial TB Program Managers based on the following criteria: 1) use of standard programmatic RR-TB treatment, 2) willingness to participate at a study site, and 3) located in the provinces of KZN and EC. Participants enrolled at five intervention sites had their care coordinated by a nurse case manager. Details of that intervention are described elsewhere [14]. Participants at the five control sites received care according to the South African RR-TB treatment guidelines at the time. This analysis includes a subset of those participants enrolled in the parent study who were assigned an outcome of cure, treatment complete, or LTFU based on the WHO definitions for multidrug-resistant TB outcome [6]. The parent study operationalized this WHO definition of LTFU by assigning patients an outcome of LTFU on the date that participants were without RR-TB medications for two consecutive months. Participants who transferred treatment site, died, or failed treatment in the parent study were excluded. This analytical approach is consistent with the current LTFU literature [8].

### Distance to treatment

Distance to treatment was calculated as the shortest one-way travel distance by road between the hospital where a patient received RR-TB treatment and the centroid of the ward (small administrative subdivisions within SA) in which they resided at treatment initiation. The participant’s residential ward was manually linked from their National Health Laboratory Service (NHLS) [15] record using name and date of birth from the start and end dates of their RR-TB treatment episode.

The residential location data that participants reported to NHLS was linked to its corresponding ward using the 2016 Ward Delimitation Maps available through South Africa’s Municipal Demarcation Board [16], Google Maps [17], and HereWeGo [18]. Although some participant location data was collected in the parent study, it was too geographically broad to be used in meaningful distance to treatment calculations; however, it was used to substantiate the location data extracted from NHLS lab reports. Ultimately, 95.7% of (1475 out of 1541) participants were successfully linked to their ward of residence.

After participants were linked to their ward of residence, distance to treatment from ward centroid to RR-TB treatment site was calculated using the *GeoRoute* command for Stata, which calculates travel distance using the HERE API [19, 20]. Per the recommendations of the *GeoRoute* command developers, the command was run twice and the results compared [19]. No discrepancies were identified between the results of the first and second runs. For 5% of the participants selected at random, the distances calculated with *GeoRoute* were compared to distances calculated with Google Maps using the same inputs and again no discrepancies were identified. This distribution of travel distance to each treatment site was examined, and for each outlier the location extracted from NHLS, ward of residence, and the distance calculation were verified. Distance was categorized for this analysis based on comparable prior research in SA investigating the impact of distance to drug-sensitive TB (DS-TB) referral hospitals (i.e., 0–20 km, > 20–40 km, > 40–60 km, and > 60 km) [21].

### Covariates

Covariates include age in years at the time of RR-TB treatment initiation; sex (male or female); HIV-status; education level (less than secondary school or completed secondary school); employment status (employed, unemployed, or other); housing stability (informal housing/unhoused or house/flat); residential locale (village/farm or urban/township); RR-TB treatment site; and treatment regimen received (injectable regimen, all-oral regimen, or individualized regimen). Demographic information was collected via patient report during parent study enrollment, while treatment regimen was extracted from medical records at the end of treatment. The covariates used in this analysis were chosen due to their potential impact on transportation access, road quality, and care-seeking behavior (i.e., residential locale and treatment site) or an association with LTFU demonstrated in previous research [5, 8, 22]. Additionally, inclusion of the treatment site variable controls for any impact of the parent study intervention, as randomization occurred at the treatment site level.

### Statistical analyses

We conducted a complete-case analysis (N = 1436), dropping any case with missing data points. This analytical method resulted in < 7% of participants being excluded from the reported data. Imputation was not performed because most of the missing data was within the independent variable – travel distance. When the missing distances were explored, missingness was not significantly associated with LTFU. Descriptive statistics were examined and compared across the four distance categories using ${\chi }^{2}$ for categorical variables and the Kruskal–Wallis H test for age.

Using multivariable logistic regression, we developed a ‘base model’ to analyze the relationship between distance to treatment and LTFU, compared to treatment success (i.e., cure and treatment complete). This ‘base model’ also controlled for two other covariates that we a priori decided to force into the model – treatment site and residential locale. Each of the other covariates was then individually added to this ‘base model’. Any covariate that was significant at a level of 0.05 when added to the ‘base model’ was included in the final multivariable logistic regression. With a sample size of 1436, power of 0.8, and alpha of 0.05, we can detect significant odds ratios equal to 1.5 or greater between our reference and smallest group. All statistical analyses were performed using Stata16.0.

## Results

### Study sample

The final sample consisted of 1436 people living in 481 wards, 38 local municipalities, and 14 districts across KZN and EC, South Africa. Among those participants, 821 (57.2%) were male, 1061 (73.9%) were living with HIV, and the median age was 35 (IQR: 29 – 43). The majority of people were unemployed (*n* = 789, 54.9%), living in a village or farm (*n* = 891, 62.0%), and had less than a secondary school education (*n* = 1034, 72.0%). Looking at clinical factors, 1152 (80.2%) received a standardized all-oral or injectable treatment regimen, depending upon when they enrolled in RR-TB treatment, while 284 (19.8%) received individualized treatment regimens. Ultimately, 1086 (75.6%) successfully completed RR-TB treatment and, 350 (24.4%) were LTFU.

The overall median distance traveled to receive RR-TB treatment was 40.96 km (IQR: 17.12, 63.49). Across the four distance categories, there were significant differences observed in housing stability (*p* = 0.001), residential locale (*p* < 0.001), employment status (*p* < 0.001), and treatment regimen (*p* = 0.001). Most notably, unstable housing (i.e., informal housing/unhoused) is more prevalent among those living closer to treatment, while living in a farm or village was more common in the farther distance categories. There were no significant differences in age (*p* = 0.842), sex (*p* = 0.342), HIV status (*p* = 0.939), and education level (*p* = 0.317) observed. Additional demographic information is presented in Table 1.

**Table 1:** Participant characteristics by distance category

	**Total** (*N* = 1436)	** ≤ 20 km** (*n* = 408)	** > 20–40 km** (*n* = 295)	** > 40–60 km** (*n* = 316)	** > 60 km** (*n* = 417)	***p*-value**
	n (%)					
LTFU	350 (24.4)	94 (23.0)	73 (24.7)	67 (21.2)	116 (27.8)	0.185
Median Travel Distance^a^	41.0 (17.1, 63.5)	10.8 (5.3, 15.0)	28.6 (23.6, 34.6)	50.5 (45.7, 55.5)	83.1 (69.0, 100.8)	< 0.001
Age^a^	35 (29, 43)	35 (29, 43)	35 (28, 43)	36 (28, 45)	35 (29, 44)	0.842
Male Sex	821 (57.2)	248 (60.8)	168 (56.9)	173 (54.7)	232 (55.6	0.342
Living with HIV	1061 (73.9)	302 (74.0)	217 (73.6)	230 (72.8)	312 (74.8)	0.939
Completed Secondary School	402 (27.9)	119 (29.2)	91 (30.8)	93 (29.4)	99 (23.7)	0.317
Unstable Housing	135 (9.4)	47 (11.5)	38 (12.9)	29 (9.2)	21 (5.0)	0.001
Employment Status						< 0.001
Employed	437 (30.4)	125 (30.6)	102 (34.6)	81 (25.6)	129 (30.9)	
Unemployed	789 (54.9)	242 (59.3)	154 (52.2)	195 (61.7)	198 (47.5)	
Other^b^	210 (14.6)	41 (10.0)	39 (13.2)	40 (12.7)	90 (21.6)	
Treatment Regimen						0.001
Injectable	786 (54.7)	209 (51.2)	148 (50.2)	177 (56.0)	252 (60.4)	
All-Oral	366 (25.5)	123 (30.1)	79 (26.8)	89 (28.2)	75 (18.0)	
Individualized	284 (19.8)	76 (18.6)	68 (23.1)	50 (15.8)	90 (21.6)	
Residential Locale (Farm/Village)	891 (62.0)	148 (36.3)	151 (51.2)	255 (80.7)	337 (80.8)	< 0.001

^a^Median, IQR, and *p*-value from Kruskal–Wallis test reported

^b^Other Employment includes homemakers, students, retirees, and people on disability

### Relationship between Distance to RR-TB Treatment and LTFU

Age, sex, HIV status, education level, employment status, and treatment regimen were selected for inclusion in the final model. In the final model, living more than 60 km from RR-TB treatment increased the odds of LTFU by 91% over those living within 20 km of RR-TB treatment (aOR: 1.91, 95% CI 1.28—2.84), when controlling for selected risk factors. Those living between > 20—40 km (aOR: 1.16, 95% CI 0.80—1.69) and > 40—60 km (aOR: 1.19, 95% CI 0.78—1.82) had slightly increased odds of LTFU, but these results were not significant. Other factors that increased risk of LTFU in the final model include male sex (aOR: 1.67, 95% CI 1.28—2.19) and living with HIV (aOR: 1.39, 95% CI 1.02—1.89). Older age (aOR: 0.98, 95% CI 0.97—0.99), completing secondary school (aOR: 0.69, 95% CI 0.51—0.93) and receiving an all-oral (aOR: 0.54, 95% CI 0.38—0.78) or individualized treatment regimen (aOR: 0.61, 95% CI 0.43—0.87) were found to protect against LTFU. (See Table 2 for full results.) Treatment site also had a significant impact on LTFU.

**Table 2:** Adjusted odds of LTFU during RR-TB treatment compared to successful outcome

	**Base Multivariable Model**^a^		**Final Multivariable Model**^a^	
	aOR	95% CI	aOR	95% CI
Travel Distance				
≤ 20 km	Ref		Ref	
> 20–40 km	1.10	0.76—1.59	1.16	0.80—1.69
> 40–60 km	1.09	0.72—1.65	1.19	0.78—1.82
> 60 km	1.84*	1.25—2.71	1.91*	1.28—2.84
Residential Locale (Farm/Village)	0.71	0.50—1.01	0.70	0.48—1.00
Age			0.98*	0.97—0.99
Male Sex			1.67*	1.28—2.19
Living with HIV			1.39*	1.02—1.89
Completed Secondary School			0.69*	0.51—0.93
Employment Status				
Employed			Ref	
Unemployed			1.19	0.88—1.60
Other^b^			0.65	0.40—1.05
Treatment Regimen				
Injectable			Ref	
All-Oral			0.54*	0.38—0.78
Individualized			0.61*	0.43—0.87

*Legend*: *aOR* Adjusted odds ratio, *Ref* Reference, *CI* Confidence interval; *p < 0.05

^a^These models also control for RR-TB treatment site

^b^Other Employment includes homemakers, students, retirees, and people on disability

## Discussion

This analysis examined the relationship between distance traveled to RR-TB treatment and LTFU in the KZN and EC, South Africa. South Africa has made great strides in decentralizing RR-TB, transitioning to an ambulatory care model for RR-TB and increasing the number of RR-TB treatment initiation sites from 17 to 658 [9]; however, KZN and EC still have the least decentralized RR-TB services in SA [9]. This analysis demonstrates that one-quarter of patients in these provinces must travel at least 60 km to receive RR-TB care, a substantial burden when you consider the road quality and transportation access issues in SA, as well as the cost and time associated with these trips.

This analysis demonstrated that these long travel distances (> 60 km) adversely impact care retention, increasing the odds of LTFU from RR-TB care by 91%. Despite lack of RR-TB research on this topic, these findings are congruent with older research demonstrating poor outcomes (i.e., death) for patients traveling long distances (> 60 km) to receive DS-TB treatment at district hospitals, which was conducted when DS-TB diagnosis and intensive phase treatment still took place at more centralized sites [21]. Research conducted within other African countries also links long travel distance to decreased TB treatment success [23, 24], increased death during TB treatment [25], and TB treatment delay [26]; however, the findings on LTFU are mixed. Despite the conflicting findings about distance and LTFU in Africa, the preponderance of the global research demonstrates a relationship between increased distance to TB treatment and decreased patient engagement [27–30]. Further, this data is supported by numerous qualitative studies in which people with TB indicated that increased distance from care and travel costs negatively impacted engagement, adherence, and care retention [12, 31–33].

Our analysis reinforces the current, but limited, data demonstrating that increased travel distance to treatment is linked to poor TB outcomes, regardless of drug-resistance pattern. One potential way to intervene on this relationship within South African RR-TB treatment facilities is more frequent down referral to and care coordination with primary healthcare facilities (PHCs). PHCs are available within 5 km of 90% of South Africans, allowing people with RR-TB to receive treatment closer to home [34]. Based on current decentralization policies, monitoring of RR-TB treatment is within the purview of PHCs [9]. Newer, short-course regimens, like BPaL [35], offer hope for greater integration within the PHC clinics. Efforts focused on building the capacity of the healthcare workers at PHCs, particularly nurses [36, 37], to effectively monitor RR-TB treatment would allow SA to capitalize on existing infrastructure to bring RR-TB closer to patients and improve treatment outcomes.

Beyond increasing geographic access, other covariates provide insight into patient engagement in care. The specific site where someone received treatment impacted their odds of LTFU. Discussions with the parent study staff revealed additional site level characteristics that may have impacted LTFU. Most notably, differences in treatment site accessibility via bus or taxi routes were hypothesized to have impacted LTFU, an important topic for future patient engagement research.

The transition away from injectable regimens has led to a notable reduction in the odds of LTFU, consistent with the initial studies reporting the outcomes of all-oral RR-TB regimens in SA [5]. All-oral regimens eliminated the need to travel to health facilities daily for aminoglycoside injections, likely decreasing the impact of travel distance on care outcomes. Most individualized regimens were the result of side effects from or contraindications to injectables which may explain why individualized regimens also reduced the odds of LTFU.

Among the nonmodifiable risk factors, we continue to see that younger males are at increased risk for LTFU, making them potential targets for interventions that promote retention [8]. The relationship between HIV and LTFU is less clear cut. Although our analysis showed HIV increased risk for LTFU, past research on this relationship is mixed [8, 38]. In populations with high rates of RR-TB/HIV coinfection, increased regimen complexity, additional side effect burden [39], and the cumulative impact of HIV and TB stigma may contribute to poorer retention of people living with HIV in RR-TB care [40]. Finally, this study links completion of secondary school with decreased odds of LTFU. Such a relationship between education level and LTFU has been seen in other geographic areas [22, 41], but not in recent studies within South Africa [42, 43]. Although there is limited ability to modify the education level of adults in treatment for RR-TB, there is potential to provide disease- and treatment-specific education. Such educational interventions may decrease LTFU, as better TB-specific knowledge has been shown to decrease LTFU in other areas of the globe [44].

One major limitation of this study is varying ward sizes. Because ward size is based on population, wards that are less densely populated are geographically larger. As a result, ward centroids are a less accurate proxy for residential locations in larger wards. In future studies, we recommend the collection of geolocations for residences. This data is important for more accurate distance-to-treatment analyses, and may also be useful in tracking patients who are lost from care, which is why it was initially recommended in the first *Policy Framework on Decentralisation and Deinstitutionalized Management for South Africa* [45]. Additionally, we were unable to capture transit type, taxi/bus routes, transit time, transit cost, and migration/movement throughout treatment, which may more accurately represent the experience of traveling to and from RR-TB treatment than simple distance. Finally, there were not enough sites to use a multilevel modeling approach, but treatment site was controlled for in the analysis. Despite these limitations, this is still one of the first analyses to characterize distance traveled to receive RR-TB treatment in SA and link it to LTFU, thus supporting the importance of continued decentralization efforts.

## Data Availability

Data cannot be shared publicly because it contains protected health information, and due to IRB confidentiality and data sharing restrictions; however, data can be made available for researchers who meet the criteria for access to confidential data. Please contact the Center Manager for The Johns Hopkins Center for Infectious Disease and Nursing Innovation, Kelly Lowensen (klowens1@jhu.edu), for additional assistance accessing study data.

## Abbreviations

BPaL: Bedaquiline, pretomanid, and linezolid
DS-TB: Drug-sensitive tuberculosis
DR-TB: Drug-resistant tuberculosis
EC: Eastern cape
HIV: Human immunodeficiency virus
km: Kilometer
KZN: KwaZulu-natal
LTFU: Loss to follow-up
NHLS: National health laboratory service
PHC: Primary healthcare facilities
RR-TB: Rifampicin-resistant tuberculosis
SA: South Africa
TB: Tuberculosis
WHO: World Health Organization
XDR-TB: Extensively drug-resistant tuberculosis
